# MicroRNA-124 Overexpression in Schwann Cells Promotes Schwann Cell-Astrocyte Integration and Inhibits Glial Scar Formation Ability

**DOI:** 10.3389/fncel.2020.00144

**Published:** 2020-07-02

**Authors:** Zhijun Li, Yifei Yu, Juanjuan Kang, Yangyang Zheng, Jinying Xu, Kan Xu, Kun Hou, Yi Hou, Guangfan Chi

**Affiliations:** ^1^The Key Laboratory of Pathobiology, Ministry of Education, College of Basic Medical Sciences, Jilin University, Changchun, China; ^2^Department of Neurosurgery, The First Hospital of Jilin University, Changchun, China; ^3^Department of Regeneration Medicine, School of Pharmaceutical Sciences, Jilin University, Changchun, China

**Keywords:** spinal cord injury, Schwann cell, microRNA-124, astrocyte, boundary, integration, glial scar

## Abstract

Schwann cell (SC) transplantation is a promising approach for the treatment of spinal cord injury (SCI); however, SC grafts show a low migratory capacity within the astrocytic environment, which inevitably hampers their therapeutic efficacy. The purpose of this study was to explore mechanisms to modify the characteristics of SCs and astrocytes (ASs), as well as to adjust the SC-AS interface to break the SC-AS boundary, thus improving the benefits of SCI treatment. We observed that the expression levels of miR-124 in SCs and ASs were significantly lower than those in the normal spinal cord. Furthermore, overexpressing miR-124 in SCs (miR-124-SCs) significantly inhibited gene and protein expression levels of SC-specific markers, such as GFAP and Krox20. The expression of neurotrophic factors, *Bdnf* and *Nt-3*, was up-regulated in miR-124-SCs without affecting their proliferation. Further, the boundary assay showed an increased number of miR-124-SCs that had actively migrated and entered the astrocytic region to intermingle with ASs, compared with normal SCs. In addition, although Krox20 protein expression was down-regulated in miR-124-SCs, the luciferase assay showed that *Krox20* is not a direct target of miR-124. RNA sequencing of miR-124-SCs revealed seven upregulated and eleven downregulated genes involved in cell migration and motility. Based on KEGG pathway and KOG functional analyses, changes in these genes corresponded to the activation of Hippo, FoxO, and TGF-beta signaling pathways, cytokine-cytokine receptor interactions, and the cell cycle. Finally, co-culturing of miR-124-SCs and ASs in a transwell system revealed that GFAP and p-STAT3 protein expression in ASs was significantly reduced. Collectively, these results show that overexpression of miR-124 in SCs promotes SC-AS integration *in vitro* and may attenuate the capacity of ASs to form glial scars. Thus, this study provides novel insights into modifying SCs by overexpressing miR-124 to improve their therapeutic potential in SCI.

## Introduction

Spinal cord injury (SCI) is a traumatic disease with direct and indirect causes. It has received increasing attention in the medical community due to its high potential of leading to disability, the difficulty of spinal cord surgery, and its impaired healing process. SCI is accompanied by a series of complex pathological changes and microenvironment imbalances that occur at the cellular, and molecular levels (Fan et al., 2018), including the formation of glial scar, which primarily consists of reactive ASs. Following SCI, ASs proliferate and become hypertrophic and reactive (Sofroniew and Vinters, 2010), migrating along, and toward, the lesions to form a dense glial scar that protects the normal tissue from inflammation and necrosis (Faulkner et al., 2004). However, the scar interlaces, resulting in steric hindrance and production of inhibitory molecules, such as chondroitin sulfate proteoglycans, which hinder the axonal sprouting and interfere with damage repair (O’Shea et al., 2017). In short, scar formation restricts tissue repair. Thus, reducing the formation of glial scar and promoting axon growth to effectively connect distal and terminal axons and fill the cavity, are vital strategies for SCI treatment.

Cell transplantation therapy is a promising treatment for SCI (Lin et al., 2012; Assinck et al., 2017). SCs are the main glial cells of the PNS derived from the neural crest. They secrete neurotrophic factors to protect neurons, strengthen axon myelination, and form Büngner bands to guide axons during peripheral nerve damage (Saberi et al., 2011; Jessen et al., 2015) evaluated the safety of autologous SC after intramedullary injection on 33 patients for 2 years and reported no incidence of deep infection or deformity. Furthermore, the phase I clinical trial of autologous human SC transplantation in subjects with SCI was approved in 2016 (Anderson et al., 2017), which provided evidence for the safety and efficacy of SC transplantation for SCI treatment.

However, its potential application has been severely restricted as SC grafts rarely intermingle with host ASs both *in vivo* and *in vitro* following injection into the host spinal cord (Andrews and Stelzner, 2007; Pearse et al., 2007; Afshari et al., 2011). Briefly, ASs insulate SCs from the CNS and hinder SC remyelination of demyelinated axons (Blakemore et al., 1986). During CNS damage, the ASs are activated, and up-regulate the expressions of GFAP (Shields et al., 2000), inhibitory molecules such as chondroitin sulfate proteoglycans (Silver, 2016), and *N*-cadherin (Fairless et al., 2005), which characterizes the interface of SCs and ASs (SC-AS), indicating a limited functional SCI recovery after SC transplantation due to the poor migration and integration between SC-AS via an unknown mechanism. Currently, migration of SCs into the AS-containing region has been effectively enhanced by magnetizing SCs with superparamagnetic iron oxide nanoparticles (Xia et al., 2016).

Accumulating evidence has demonstrated that artificially altering SC intrinsic micoRNA (miRNAs) levels may serve to modify inherent cells’ characteristics and potentially promote SC-AS integration. miRNAs are a class of non-coding small endogenous RNA molecules (20–24 nucleotides in length) that either suppress the translation of, or degrade target mRNAs, by complementary interaction with specific sequences in its 3′-untranslated regions (3′-UTR). MiRNAs are involved in nearly all biological processes, including cell proliferation, differentiation, and development. For instance, the transfection of SCs with the miR-221/222 mimic was reported to promote their proliferation and migration following sciatic nerve damage (Yu et al., 2012). Additionally, miR-sc8 inhibits SC proliferation and migration by targeting Egfr (Gu et al., 2015), indicating that the artificial manipulation of miRNAs in SCs alters the biological characteristics of the latter. In the present study we focused on microRNA-124 (miR-124), which is a non-coding miRNA ubiquitous in the brain and plays important roles in neurogenesis and neuronal maturation, while also modulating neural stem cell differentiation (Sun et al., 2015). MiR-124 low-expressing cells, such as Hela, fibroblasts, embryonic stem cells, and mesenchymal stem cells, become induced to differentiate into neurons via upregulating miR-124 levels (Lim et al., 2005; Ambasudhan et al., 2011; Mondanizadeh et al., 2015). Moreover, our preliminary study demonstrated relatively low expression of miR-124 in ASs cultured from neonatal rat cortices, and decreased Sox9 and GFAP expression following overexpressing miR-124 (unpublished data). These results demonstrated that in cells with low miR-124 levels, its ectopic expression alters inherent cellular characteristics. We also observed in our preliminary studies that SCs express lower miR-124 levels than neural tissue. Therefore, in this study, we hypothesized that by enhancing miR-124 levels, the inherent characteristics, bioactivities, and motility of SCs would be modulated to ultimately promote SC-AS integration. However, the effect of miR-124 levels on SCs has not yet been previously reported. To the best of our knowledge, this is the first study to assess the effects of miR-124 overexpression on SCs differentiation.

In this study, we modified the characteristics and bioactivities of SCs using a specific miRNA to promote the integration of SC-AS. We showed that the overexpression of miR-124 in SCs facilitates SC-AS integration by indirectly down-regulating Krox20 expression and inhibiting the activation of ASs. Our novel findings could serve as theoretical guidance to improve the interface between SC grafts and host ASs, thus reducing the occurrence of astrogliosis. Hence, SCI treatment using miR-124-modified SCs may strengthen neural tissue repair, thus providing a therapeutic benefit.

## Materials and Methods

### Antibodies and Reagents

The following antibodies were used in this study: anti-Sox10 Rabbit antibody (69661; CST, United States); anti-GFAP rabbit antibody (ab7260; Abcam, United Kingdom); anti-NGFR/p75 NGF Receptor mouse antibody (MLR2; Thermo, United States); anti-GAPDH mouse antibody (AT0002; CMC-TAG, United States); anti-β-actin mouse antibody (AT0001; CMC-TAG); anti-Sox9 rabbit antibody (ab5535; Abcam); Goat anti-rabbit IgG H&L (Allexa 488/594; Abcam); anti-S100β rabbit antibody (ab52642; Abcam); anti-EphA4 rabbit antibody (GTX1116; GeneTex, United States); anti-N-Cadherin rabbit antibody (GTX1273; GeneTex); and anti-Krox20 rabbit antibody (ab108399; Abcam).

We used the following cytokines: human FGF basic (bFGF, Peprotech, United States), human-heregulin (neuregulin) β-1 (Peprotech), Forskolin (Selleckchem, United States), and N2-supplement (100×; Gibco, United States), TGF-β1 (Peprotech).

The biological reagents and kits used are as follows: RNeasy Mini Kit (Qiagen, Germany), All-in-One First-Strand cDNA Synthesis Kit (GeneCopoeia, United States), TransScript One-step gDNA Removal and cDNA Synthesis SuperMix (Transgen, China), 2× Easytaq PCR SuperMix (+Dye) (Transgen), ChamQ Universal SYBR qPCR Master Mix (Vazyme, China), All-in-One miRNA qRT-PCR Reagent Kits (GeneCopoeia), Deoxyribonuclease I (Worthington, United States), DMEM/F-12 (1×) (Hyclone, United States), trypsin 0.25% EDTA (1×) (Gibco, United States), trypsin 0.05% EDTA (1×) (Gibco), type I Collagenase (Sigma, United States), BeyoECL Plus (Beyotime, China), PBS (Solarbio, China), FBS (Transgen), poly-D-lysine hydrobromide (Sigma), penicillin-streptomycin liquid (Invitrogen, United States), tritonX-100 (Solarbio), Hoechst 33342 (Beyotime, China), agarose (Biowest, France), RIPA lysis buffer (Solarbio), and 4% PFA (Solarbio).

### Culture of SCs and Astrocytes

All animals were supplied by the Experimental Animal Center (Yisi Company, China). All animal experimental procedures were approved by the Ethics Committee of Jilin University and performed in accordance with the institutional guidelines (ethical approval code: 2018-12).

Primary SCs were prepared from the sciatic nerves of adult female Wistar rats (200–220 g) as previously described with minor modifications (Andersen et al., 2016). Briefly, the sciatic nerves were removed from the epineurium using fine forceps under the microscopic examination. They were then sliced (2 mm long) and digested with 0.1% type I collagenase in DMEM/F-12 for 20 min at 37°C under 5% CO_2_ atmosphere. Next, the tissue samples were rinsed with PBS, carefully seeded onto a sterile cell culture dish, and cultured in DMEM/F-12 with 10% FBS. The media was changed every 3 days. Many fibroblasts migrated out from the tissue samples during this period. Approximately every 12 days, the tissue samples were transferred to a new culture dish, which was repeated until fibroblast migration from the tissue samples ceased and bipolar SCs migration increased; generally requiring 3–4 weeks. At which point the medium was changed to the Schwann cell medium (ScM) containing DMEM/F-12, 10% FBS, forskolin (2.5 μM), bFGF (10 ng/mL), and neuregulin-β1 (15 ng/mL) every 3 days for 1 month to facilitate active proliferation of SCs. Afterward, the tissue samples were homogenized with a 5 mL pipette, collected in a 15 mL centrifuge tube, and centrifuged for 5 min (1,500 rpm). The supernatant was discarded, and the remaining tissue was digested with 0.25% trypsin for 5 min at 37°C. Enzymatic treatment was stopped once a single-cell suspension was obtained. Cells were plated onto a poly-D-lysine (Sigma)-coated 10 cm^2^ cell culture dish and maintained with ScM.

Primary ASs were prepared from the cerebral cortex of postnatal Wistar rats (days 1–3). The vascular membrane and excess cortices were removed and digested with 0.05% trypsin and deoxyribonuclease I (4 ng/mL) for 25 min at 37°C. Enzymatic treatment was stopped with 10% FBS supplemented DMEM/F-12; the solution was filtered through a 40 μm sterile EASYstrainer (Greiner Bio-One, Germany) to achieve single-cell suspension, and cells were collected after centrifugation. The cells were then seeded into cell flasks and cultured in the AS medium containing DMEM/F-12 with 10% FBS, bFGF (10 ng/mL), TGF-β1 (10 ng/mL), and N2-supplement (1×). After the cells proliferated to confluency, unattached cells including microglial and oligodendrocytes were removed by shaking continuously at 200 rpm for 8 h at 37°C daily for 2 days.

### Immunocytofluorescence

Confluent cells were fixed with 4% PFA (Solarbio) for 30 min and washed with PBS. Unspecific binding was blocked with 0.2% triton X-100 and 10% goat serum for 45 min. Primary antibodies (p75^*NTR*^ 1:200, GFAP 1:1000, Sox10 1:1600, S100β 1:100) were diluted in 1% goat serum and incubated with the cells overnight at 4°C. The cells were rinsed with PBS and incubated with secondary antibodies while shielded from light for 1 h at room temperature. Samples were washed with PBS and stained with Hoechst 33342 for 10 min and stored in PBS. The cells were studied under a fluorescence microscope (Olympus IX71, Germany), and the images were captured by cellSens Entry Software.

### Construction of Lentiviral Vectors and Transfection of SCs

The linearized vector was obtained through restriction digestion. PCR was performed to amplify the vector. The final sequences of the 5’ and 3’ amplification products were consistent with the terminal sequences of the linearized vector. The reaction system was prepared with the linearized vector and the objective amplification products for recombination reaction. LV-rno-mir-124-1 (Genechem, China) was constructed using GV309, pHelper 1.0 (*gag*, *pol*, and *rev*), and pHelper 2.0 (*VSV-G*) plasmids. Egr2-RNAi (Genechem) was constructed using GV102 plasmids inserted with shRNAs (*Egr2-RNAi* 6164-1, *Egr2-RNAi* 6165-11, and *Egr2-RNAi* 6166-1). The plasmids were transfected into 293T cells and cultured for 48 ∼ 72 h. The supernatant was harvested and filtered, and the recombinant lentiviral vector containing the GFP reporter gene was obtained (Genechem). SCs were seeded in a 24-well plate and transfected with lentiviral vectors with the *miR-124* or *Egr2-RNAi* sequence at a MOI. The medium was exchanged with the fresh one after transfection for 18 h, and the strongest GFP expression was detected 48 h after transfection. The transfection efficiency of the lentiviral vectors with the *miR-124* and *Egr2-RNAi* sequences was evaluated via quantitative RT-PCR and western blotting, respectively.

### Reverse Transcriptase-Polymerase Chain Reaction

Total RNA was extracted from the control cells using QIAzol (Qiagen), and the concentration was measured by NanoDrop 2000c (Thermo, United States). Genomic DNA was removed using the TransScript One-step gDNA Removal, and RNA was reversed transcribed (RT) in a reaction containing 500 ng total RNA, 1 μL Anchored Oligo (dT)_18_ primer (0.5 μg/μL), 10 μL o2 × TS Reaction Mix, 1 μL TransScript RT/RI Enzyme Mix, and 1 μL gDNA Remover from the cDNA Synthesis SuperMix kit (Transgen). Double distilled water was added to the reaction to a final volume of 20 μL. The products were incubated for 30 min at 42°C for RT reaction; then TransScript RT/RI Enzyme and gDNA Remover were inactivated for 5 s at 85°C.

The cDNA was amplified by PCR in a reaction containing 0.8 μl cDNA, 0.4 μL Forward Primer, 0.4 μL Reverse Primer, 10 μL 2× Easytaq PCR SuperMix (+Dye), and 8.4 μL double distilled water. The samples were initially incubated at 94°C for 5 min, followed by 35 cycles of denaturation at 94°C for 30 s, annealing at primer-specific temperature for 30 s, and extension at 72°C for 30 s; the final extension was performed at 72°C for 7 min. The PCR products were electrophoresed in a 1.5% agarose gel (Applied Biosystems, United States) and analyzed with a Gel Image System (Tanon, China). The figures were captured by Tanon MP software and analyzed by ImageJ. The primer names and sequences used are listed in Table 1.

**TABLE 1:** Primer sequences used for RT-PCR.

Gene name	Forward sequences (5′ → 3′)	Reverse sequences (5′ → 3′)
*GFAP*	GGTGGAGAGGGACAATCTCA	TGTGAGGTCTGCAAACTTGG
*Sox10*	CCAGGCTCACTACAAGAGTGC	CCTGTGGTCTCTGTCTTCACC
β*-actin*	GCTGTGTTGTCCCTGTATGC	GAGCGCGTAACCCTCATAGA

### Quantitative RT-PCR

We analyzed the relative expression of miR-124 in primary SCs, ASs, normal spinal cord, miR-124-SCs, and SCs in NC. RNA was extracted with QIAzol (Qiagen) and subjected to RT reaction with All-in-One First-Strand cDNA Synthesis Kit (Genecopoeia). Later, the RT reaction was conducted with the Applied Biosystems 7300 Plus Real-time PCR System software. The reaction contained approximately 100–1,000 ng total RNA, 1 μL 2.5 U/μL poly-A polymerase, 1 μL RTase Mix, 5 μL 5× PAP/RT buffer, and double distilled water to a final volume of 25 μL. The RT conditions were as follows: 60 min at 37°C, 5 min at 85°C, and extension at 4°C.

The qPCR reaction contained 10 μL 2× All-in-One qPCR Mix, 2 μL cDNA (100–1,000 ng, diluted 1:5), 0.4 μL ROX Reference Dye, 2 μL miR-124 forward primer, 2 μL Universal Adaptor PCR Primer (2 μM), and 3.6 μL double distilled water. The reaction was conducted as follows: pre-denaturation at 95°C for 10 min and 40 cycles of denaturation at 95°C for 10 s, annealing at 60°C for 20 s, and extension at 72°C for 10 s. U6 snRNA was the internal reference control used, and the miR-124 expression was quantified by the 2^–ΔΔ*Ct*^ method. The experiment was repeated at least thrice.

SC secretion of neurotrophic factors, such as ciliary neurotrophic factor (*Cntf*), neurotrophin-*3* (*Nt-3*), brain-derived neurotrophic factor (*Bdnf*), and nerve growth factor (*Ngf*), was analyzed following transfection. Total RNA extracted from SCs 48 h after transfection was used to synthesize cDNA using TransScript One-step gDNA Removal and cDNA Synthesis SuperMix. Afterward, quantitative RT-PCR was performed with ChamQ Universal SYBR qPCR Master Mix and the Applied Biosystems 7300 Plus Real-time PCR System. The reaction volume contained 10 μl ChamQ Universal SYBR qPCR Master Mix (2×), 0.4 μL forward primer (10 μM), 0.4 μL reverse primer (10 μM), 2 μL cDNA, and 7.2 μL double distilled water. The reaction was performed as follows: pre-denaturation at 95°C for 30 s, 40 cycles of denaturation at 95°C for 10 s, and annealing at 60°C for 30 s. β-actin was selected as the internal reference, and the expression of mRNAs was analyzed by the 2^–ΔΔ*Ct*^ method. The experiments were repeated at least thrice. The primer names and sequences are shown in Table 2.

**TABLE 2:** Primer sequences used for qRT-PCR.

Gene name	Forward sequences (5′ → 3′)	Reverse sequences (5′ → 3′)
*Cntf*	TTGTGTCCTGGGACAGTTGA	AGTCGCTCTGCCTCAGTCAT
*Nt-3*	AGTGTGTGACAGTGAGAGCCTGTGG	GAGAGTTGCCGGTTTTGATCTCTCC
*Bdnf*	ATGCTCAGCAGTCAAGTGCCTTTGG	GCCGAACCCTCATAGACATGTTTGC
*Ngf*	ACCCAAGCTCACCTCAGTGTCTGG	CATTACGCTATGCACCTCAGAGTGG
*GFAP*	CGAGTTACCAGGAGGCACTA	TCCACGGTCTTTACCACAAT
*Sox9*	GTGCTGAAGGGCTACGACTGGA	GTTGTGCAGATGCGGGTACTGG
*S100*β	GAGAGAGGGTGACAAGCACAA	GGCCATAAACTCCTGGAAGTC
*GAPDH*	AGACAGCCGCATCTTCTTGT	CTTGCCGTGGGTAGAGTCAT

### Western Blotting

After transfection, the SCs were incubated with RIPA lysis buffer and PMSF (RIPA: PMSF = 100: 1) in RNase tubes for 30 min in an ice bath. The samples were centrifuged for 20 min at 4°C (12,000 × g). The supernatants were collected, and the protein concentrations were measured by a Bicinchoninic Acid kit and NanoQuant plate analyzer (Infinite m200 PRO, Tecan, Switzerland). The protein samples were boiling for 5 min to denature them and were stored at −20°C until use. Western blotting was performed with 20 μg of each sample/well on a 10% polyacrylamide gel. After electrophoresis, the proteins were transferred onto PVDF membranes for 1.5 h at 100 V. Primary antibodies were incubated on the shaker for 1 h and preserved at 4°C overnight. Goat anti-mouse IgG (H + L, HRP conjugate, 1:2000) and goat anti-rabbit IgG (H + L, HRP conjugate, 1:2000) were added to the samples for 1 h at room temperature. Proteins were detected with BeyoECL Plus (Beyotime, China), and images were captured by Gel Image System (Tanon) and analyzed by ImageJ. GAPDH was used as the internal reference protein. The experiments were repeated at least thrice.

### Cell Proliferation Assay

Cell proliferation was measured using the Cell-Light EdU Apollo643 *In Vitro* Kit (EdU, Ribobio, China). SCs were transduced with lentivirus for 48 h, at which point the virus infection peaked and the SCs overexpressing miR-124 (miR-124-SCs) were established *in vitro*. They were then seeded into 96-well cell culture plates at 3 × 10^3^ cells per well and cultured until the appropriate stage. EdU medium was then added to the cells and incubated for 2 h at 37°C. The cells were fixed with 4% PFA for 30 min, washed with PBS, and incubated with 1× Apollo staining solution for 30 min in the dark. The staining solution was removed, and the cells were permeabilized with 0.5% Triton X-100. Nuclear cell staining was performed with Hoechst 33342 for 10 min. Two duplicate wells were included in each experimental group (i.e., WT, NC, and miR-124). A total of 10 fields of vision were selected for each well, and the images were obtained under fluorescence microscopy (Figure 3F). Fluorescence expressing cell numbers were quantified by ImageJ software, and the color mode of the image was converted into an 8-bit gray scale image and inverted to a black and white image by Edit/Invert. The contrast was then adjusted using Image/Adjust/Threshold. The positive rate was calculated from the total number of cells and the number of positive-staining cells. All data were analyzed using GraphPad Prism 7.0 (United States) software.

Cell proliferation was measured using the Cell Counting Kit-8 (CCK-8, Dojindo, China). After transfection for 48 h, 96 h, and 7 days, SCs were seeded into 96-well cell culture plates at 2 × 10^3^ cells per well and cultured to the appropriate stage. CCK-8 medium was added to the cells and were incubated at 37°C for 4 h. The absorbance of each well at 450 nm was analyzed with a NanoQuant plate analyzer (Infinite m200 PRO, Tecan).

### Boundary Assay

Boundary assay was performed according to the method by Afshari with some modifications (Afshari et al., 2011; Afshari and Fawcett, 2012). SCs and ASs were harvested and centrifuged for 5 min. A straight line in reverse was carved in the cell culture plate with a diamond knife. Posteriorly, 20 μL SC suspension (1 × 10^5^/20 μL) was dropped on one side of the line in the well and was smeared toward the edge of the line with a sterile glass strip; then, 20 μL ASs suspension (3 × 10^4^/20 μL) was dropped on the opposite side of the line and spread with the glass strip parallel to the SCs, leaving a small gap between them. The cells were cultured at 37°C for 40 min. Then, the non-attached cells were removed with DMEM/F-12, whereas the adhered cells were cultured with DMEM/F-12 containing 10% FBS. The culture was maintained for approximately 5 days in a 5% CO_2_ incubator at 37°C until the two cell fronts reached each other. The plate was kept for another 7 days in the incubator, and then the cells were fixed with 4% PFA. Unspecific binding of antibodies was blocked by incubating the fixed cells with 0.2% triton X-100 for 30 min, followed by 10% goat serum for 45 min. ASs were incubated with primary antibody (GFAP 1:1000) diluted in 1% goat serum overnight at 4°C. SCs displayed green autofluorescence after transfection (lentiviral plasmids carried a GFP reporter gene).

Boundary images were captured and analyzed and fluorescence was quantified as follows. To determine the compatibility of integration, the maximum distance and relative migration areas of SCs and ASs were compared, and the interface between the confluence of SCs and ASs was considered as the baseline. Lentiviral transfection with the control sequence was set as the NC group. The cell migration distance was measured using the scale provided with the electronic software of the fluorescence microscope. A minimum of ten fields of vision were selected for each group to obtain the maximum distance. NC was set as 1, the multiple of the experimental group higher than NC was then calculated and appropriate statistical analysis was performed. The cell migration area was quantitatively analyzed by ImageJ software. A minimum of 10 fields of view were also selected to analyze the cell migration area of each group. The color mode of each image was converted to the black and white mode under the GRB stack, the shape of the red area was adjusted closest to the fluorescent area of the original image under Adjust/Threshold, and finally the fluorescent area was calculated using the result obtained by Set measurement/Area fraction. The multiple of the migration area in NC was then calculated and the appropriate statistical analyses performed. Each experiment was repeated a minimum of three times.

### Transwell Assay

To test the indirect effect of miR-124 overexpression of SCs on ASs and to simulate the three-dimensional (3D) microenvironment after transplantation, Transwell assays were performed with 6-well Polyester membrane Transwell Clear inserts (Corning, 3450; pore size: 0.4 μm). SCs were transfected by lentiviral vectors comprising miR-124 and GFP sequences for 72 h, and they were plated into the upper chamber at a density of 3 × 10^5^ cells per well in the presence of 1.5 mL DMEM/F-12 containing 10% FBS. ASs were cultured into the lower chamber filled with 2.6 mL DMEM/F-12 containing 10% FBS. The cultures were maintained for 7 days, and the media were refreshed every 3 days. RNAs and proteins were extracted from SCs and ASs for further analyses.

### Dual Luciferase Assay

*Krox20* (also known as *EGR2*) was predicted as the target gene of miR-124 using the Targetscan^1^. The miR-124 binding site in the 3′-UTR of *Krox20* mRNA and the fragments harboring the mutant miR-124 binding site were inserted into the luciferase reporter gene plasmid to obtain 3′UTR, 3′UTR-NC, and 3′UTR-MU plasmids, respectively. The 293T cells were cultured and transfected with plasmids constructed with the *hU6-MCS-CMV-GFP-SV40-Neomycin* (Rat, MI0000893, Genechem) (Table 3) and X-tremeGENE HP transfection reagent (Roche, Switzerland). *TRAF6* and *has-mir-146b* were used as the positive control (Li et al., 2013; Park et al., 2015). Transfection efficiency after 48 h was determined by measuring the fluorescence emitted by the labeled genes contained in the plasmid under a fluorescence microscope. Luminescence was measured with the microplate reader. Comparison between *3*′*UTR-NC* + *miR-124*, *3*′*-UTR* + *miR-124*, and *3*′*UTR-MU* + *miR-124* was based on the same miRNA (miR-124) plasmid, which was calibrated to eliminate differences, and re-quantified using the Firefly/Renilla luminescence fold comparison to determine whether the *3*′*UTR-NC*, *3*′*UTR*, and *3*′*UTR-MU* plasmids combined with miRNA to hinder the expression of luciferase.

**TABLE 3:** Various plasmids used in dual luciferase assay.

Plasmid names	Description
miRNA-NC	Empty plasmid used as negative control for miRNA-124
miRNA	Rno-miR-124-1 (53852-4)
3′UTR-NC	Empty plasmid used as negative control for 3’UTR
3′UTR	3′UTR for Egr2 (53866-1)
3′UTR-MU	3′UTR mutant for Egr2 (53867-1)
Positive control-3′UTR	3′UTR for TRAF6
Positive control-miRNA	Has-miR-146b

### RNA Sequencing

RNA sequencing was performed by Sangon Biotech (MRNAA191168CC, Sangon Biotech, China). RNA from the SCs transfected with miR-124-containing lentiviral vectors and its respective control (negative for miR-124) was extracted with Qiazol (Qiagen). RNA quantity control and sequenced reads were performed with FASTQ. Mapping statistics were applied with HISAT2, and the duplicated reads were obtained from the read-distribution of insert gene of RSeQC output. The distribution of gene coverage and gene density in the chromosome were analyzed by BEDTools. Homogeneous distribution was performed with Qualimap. Single nucleotide polymorphisms (SNP)/InDel calling were detected by VCF tools to find the mutated regions. Genetic structure analysis included ASprofier for alternative splicing (AS) and EricScript for fusion analysis. Gene expression was analyzed and explored by StringTie and WGCNA. Analysis of differentially expressed genes (DEG) was performed with DESeq2. Meanwhile, the results were visualized as scatter, volcano, and MA plots. To obtain significantly different genes, we set the following conditions: *Q*-value < 0.05, and | Foldchange| > 2. TopGO was used for gene ontology (GO) enrichment analysis, and the significant GO-directed acyclic graph was plotted. ClusterProfiler was applied for analysis of the Kyoto encyclopedia of genes and genomes (KEGG) pathway and eukaryotic ortholog groups (KOG) classification enrichment. The network analysis was performed based on the results of gene function enrichment analysis.

### Statistical Analysis

Data from western blotting, RT-PCR, qRT-PCR, and the EdU test were analyzed using GraphPad Prism 7.0 (United States) software. Comparisons of these results (number of experimental groups ≥ 3) were determined by one-way analysis of variance (ANOVA). The normality of data was determined by the Shapiro–Wilk normality test. Data was of normal distribution when *p*-value > 0.1. The Kruskal–Wallis test was applied when data did not pass the normality test. The results of CCK-8, western blotting for plasmid transfection, and the boundary assay involved two sets of comparative experiments and were analyzed by unpaired *t*-test (two-tailed); all of which were normally distributed (*p* > 0.1). The results of the dual luciferase assay were analyzed using an unpaired Student’s *t*-test; differences between *3*′*UTR-NC* + *miR-124/3*′*UTR* + *miR-124*, *3*′*UTR* + *miR-124/3*′*UTR-MU* + *miR-124*, and *TRAF6-3*′*UTR* + *miR-NC/TRAF6-3*′*UTR* + *has-miR-146b* were detected.

*p*-values corresponding to ^∗^*p* < 0.05, ^∗∗^*p* < 0.01, ^∗∗∗^*p* < 0.001, and ^****^p < 0.0001 were considered statistically significant. All data are presented as the mean ± SEM. Power analysis of all data was performed using G^∗^power 3 (Faul et al., 2007), and the results are shown as power (1−β) > 0.8.

## Results

### Immunocytofluorescence Confirms the Isolation of Schwann Cells and Astrocytes

**FIGURE 1:**
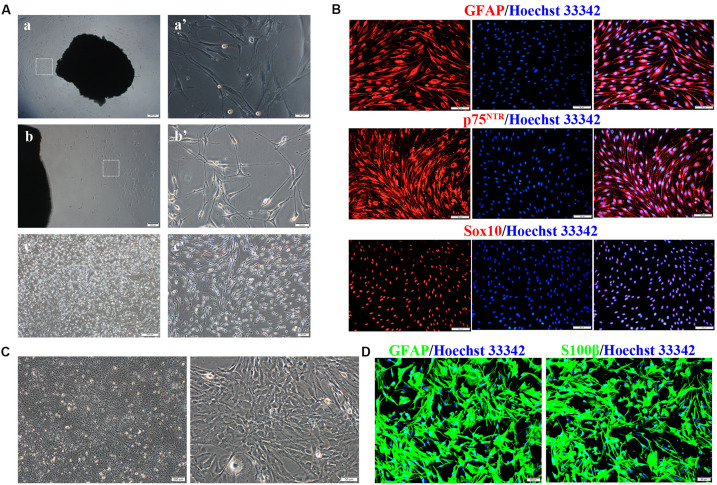
Isolation, culture, and identification of Schwann cells (SCs) and astrocytes (ASs). **(A)** Cultivation of SCs from sciatic nerve tissue. **(a,a′)** Numerous fibroblasts proliferated on day 10 of tissue culture, while few SCs survived. **(b,b′)** SCs reached a high confluence on day 30 of culture and were digested. **(c,c′)** The primary SCs were elongated in a spindle shape. The right graph is the magnification of the white dotted box of the left graph (**a–c**, scale bar = 200 μm; **a′–c′**, scale bar = 50 μm). **(B)** Characteristic markers (GFAP, Sox10, p75^*NTR*^) were highly expressed in SCs (red). The nucleus was dyed by Hoechst 33342 (blue) (*n* = 3, scale bar = 50 μm). **(C)** ASs derived from the neonatal rat brain cortex, presenting a branched, irregular morphology (left figure, scale bar = 200 μm; right figure, scale bar = 50 μm). **(D)** Characteristic markers (GFAP, S100β) were highly expressed in ASs (red), and the nuclei were stained by Hoechst 33342 (blue) (*n* = 3, scale bar = 50 μm).

Primary SCs were prepared from the sciatic nerve of adult Wistar rats (Figure 1A). Several fibroblasts proliferated and migrated from the tissue on day 10 of culture (Figures 1a,a′). SCs proliferated rapidly and reached high confluency in ScM on day 30 of culture (Figures 1b,b′). Their morphology can be described as needle-like, spindle-shaped, bipolar or triple-polar with elongated and spiral growth, which corresponds to normal SCs after digestion and purification (Figures 1c,c′). Immunocytochemistry detected the primary SCs. The expressions of GFAP, p75^*NTR*^, and Sox10 were localized in the cytoplasm, on the cell membrane, and in the nucleus, respectively (Figure 1B). These results confirmed the successful isolation and culture of SCs.

Primary ASs were derived from the brain cortices of neonatal rats 1–3 days after birth. The cells proliferated rapidly with an irregular morphology, having numerous branches and rich cytoplasm (Figure 1C). The expressions of GFAP and S100β were detected in the cytoplasm by immunocytofluorescence staining (Figure 1D), indicating successful isolation and culture of ASs.

### Transfection of Schwann Cells With a Lentiviral Vector Overexpressed MicroRNA-124 in Schwann Cells and Astrocytes

**FIGURE 2:**
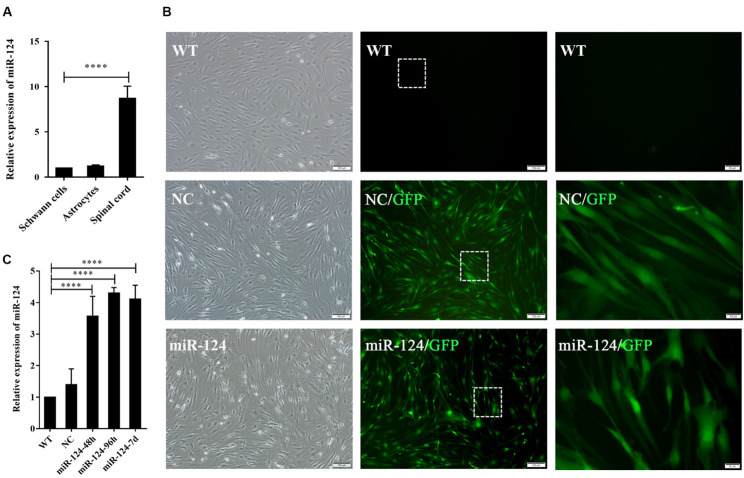
Transfection efficiency of Schwann cells (SCs) with miR-124-containing vector. **(A)** miR-124 expression in SCs and ASs was significantly lower than that in the normal spinal cord according to qRT-PCR analysis (*n* = 3, ****p < 0.0001). **(B)** SCs showed green fluorescence 48 h after transfection with lentivirus. No significant cell morphology change was noted. The third figure is the magnification of the white dotted box in the middle one (first and second, scale bar = 100 μm; third, scale bar = 20 μm). **(C)** miR-124 expression in SCs after transfection for 48 h, 96 h, and 7 days were increased fourfold than those in NC and WT (*n* = 4, ****p < 0.0001).

We next evaluated miR-124 expression in rat SCs, ASs, and normal spinal cord (isolated from adult Wistar rats). As shown in Figure 2A, the relative expression of miR-124 in SCs and ASs was remarkably lower than that in the normal spinal cord (^****^p < 0.0001).

The lentiviral vector carrying the miR-124 sequence was constructed, and its control sequence served as the NC. Untreated and non-transfected SCs were used as WT controls. The LV-rno-mir-124-1 was introduced into SCs at various MOIs (1, 10, 25, 50, and 75), and the fresh medium was changed after 18 h. Fluorescence was observed 48 h after transfection and the condition in which a minimum of 90% infection efficiency was observed, with the lowest level of cytotoxicity induced (MOI of 50) was deemed the optimal MOI for further assays. To explore the duration of transfection, we assessed the expression of miR-124 at 48 h, 96 h, and 7 days after transfection by qRT-PCR, the results of which are shown in Figures 2B,C (^****^p < 0.0001). miR-124 expression levels increased by approximately three times after transfection compared with those of the NC and WT. Therefore, the transfection method was effective and consistent. Herein, we refer to SCs with up-regulated miR-124 levels as miR-124-SCs.

### Overexpression of MicroRNA-124 Inhibited the Gene Expression of Schwann Cell-Specific Markers and Promoted the Gene Expression of Neurotrophic Factors Without Affecting Proliferation

**FIGURE 3:**
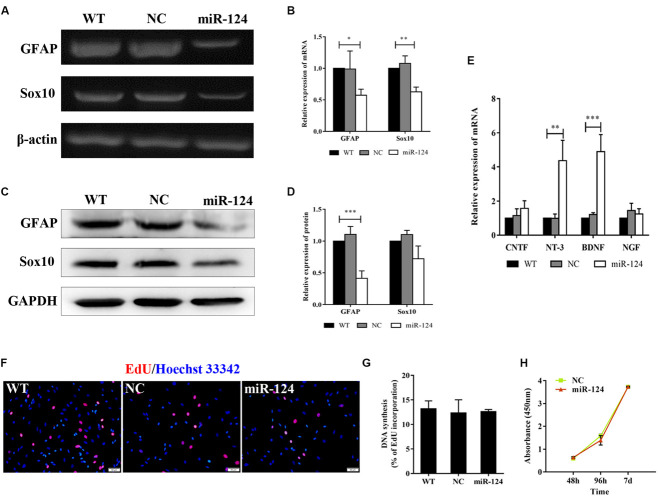
Analysis of mRNAs and protein expression levels of Schwann cell (SC) markers and their proliferation after miR-124 overexpression. **(A,B)** *GFAP* and *Sox10* mRNA levels were down-regulated as detected by RT-PCR (*n* = 3, *p < 0.05, **p < 0.01). **(C,D)** The protein expression of GFAP was significantly decreased, whereas that of Sox10 was unaffected (*n* = 3, ***p < 0.001). **(E)** The gene expressions of *Nt-3* and *Bdnf* were significantly up-regulated, whereas those of *Cntf* and *Ngf* did not significantly change (*n* = 3, **p < 0.01, ***p < 0.001). **(F–H)** SCs proliferation was not influenced by miR-124 overexpression (EdU assay: *n* = 3, *p* > 0.05, scale bar = 50 μm; CCK-8 assay: *n* = 5, *p* > 0.05).

Sox10 is a key transcription factor for myelination and differentiation, which can regulate the fate of SCs (Reiprich et al., 2010). SC-specific markers after miR-124 overexpression were investigated by RT-PCR. The expressions of *GFAP* and *Sox10* genes were significantly reduced in miR-124-SCs, compared with the NC and WT (Figures 3A,B; ^∗^*p* < 0.05, ^∗∗^*p* < 0.01). The expression of GFAP was also significantly reduced, whereas that of Sox10 did not change (Figures 3C,D; ^∗∗∗^*p* < 0.001). SCs secretion of neurotrophic factors plays an important role in nerve regeneration and neural protection. Therefore, we then evaluated the gene expressions of neurotrophic factors such as *Cntf*, *Nt-3*, *Bdnf*, and *Ngf* after transfection by qRT-PCR. The results indicated that the gene expressions of *Nt-3* and *Bdnf* were significantly up-regulated as shown in Figure 3E (^∗∗^*p* < 0.01, ^∗∗∗^*p* < 0.001).

Schwann cell proliferation after miR-124 overexpression was tested by CCK-8 and EdU assays. No significant change was observed (Figures 3F–H; *p* > 0.05). These results revealed that the overexpression of miR-124 in SCs inhibited the transcription and translation of the *GFAP* gene, as well as the transcription of *Sox10* gene. In contrast, it enhanced the gene expressions of *Bdnf* and *Nt-3* and did not affect cell proliferation.

### *In vitro* Integration of Schwann Cells and Astrocytes Was Promoted by MicroRNA-124 Overexpression

**FIGURE 4:**
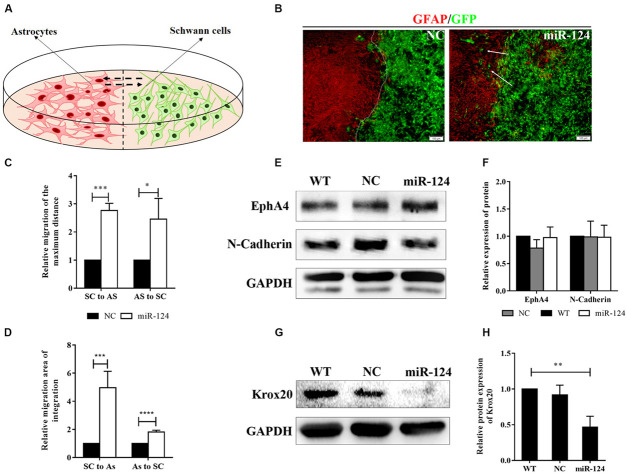
MiR-124 promoted *in vitro* integration of Schwann cells (SCs) and astrocytes (ASs). **(A)** A diagram for modeling the boundary assay for SCs and ASs. **(B)** The SC-AS integration was investigated by immunofluorescence. ASs were detected using anti-GFAP antibody (red), whereas SCs exhibited GFP (green) fluorescence. SCs migrated into astrocytic regions after miR-124 overexpression, while a sharp boundary formed between normal SCs and ASs. The white arrows indicate SCs migrating to ASs (scale bar = 100 μm). **(C)** The relative maximum distances of SC-AS migrating toward each other were both higher after miR-124 overexpression in SCs (*n* = 3, *p < 0.05, ***p < 0.001). **(D)** The relative migration areas of SCs and ASs were significantly higher after miR-124 up-regulation (*n* = 4, ***p < 0.001, ****p < 0.0001). **(E,F)** The expressions of EphA4 and N-Cadherin in SCs did not significantly change (*n* = 3, *p* > 0.05). **(G,H)** The expression of Krox20 in SCs was obviously lower after miR-124 overexpression (*n* = 4, **p < 0.01).

We then explored SC-AS integration after the up-regulation of miR-124 in SCs through the boundary assay (Figure 4A). Cells were fixed after remaining in culture for another 2 weeks, after which the two cell fronts reached each other. As shown in Figure 4B, NC cells were significantly separated and formed a sharp boundary, whereas miR-124-SCs and ASs were mixed. SCs migrated to distant areas in the astrocytic region and proliferated in combination with ASs. The SC-AS integration was quantified and compared with the maximum distance of migration and cell migration area by cellSens Entry and ImageJ software. As shown in Figures 4C,D, the relative migrations of miR-124-SCs and ASs to the maximum distance were longer than those of the control SCs and ASs. Therefore, the relative migration abilities of both cells in the experimental group have been improved (^∗^*p* < 0.05, ^∗∗∗^*p* < 0.001, ^****^p < 0.0001).

We therefore speculated that SCs migration and intermingling with ASs could be inhibited by Eph/ephrin (Afshari et al., 2010) and N-cadherin (Fairless et al., 2005). The protein expressions of EphA4 and N-cadherin in SCs after miR-124 overexpression did not significantly change (Figures 4E,F; *p* > 0.05), indicating that the integration of miR-124-SCs and ASs was not regulated by either Eph/ephrin reaction or N-cadherin inhibition.

We found that the Krox20 protein expression in SCs after miR-124 overexpression was significantly lower than those in WT and NC (Figures 4G,H), thereby illustrating that the integration of miR-124-SCs and ASs might be regulated by the down-regulation of Krox20 (^∗∗^*p* < 0.01).

### MicroRNA-124 Mediated Schwann Cell-Astrocyte Integration by Indirectly Regulating Krox20

We further explored the relationship between SC-AS integration and Krox20 expression.

**FIGURE 5:**
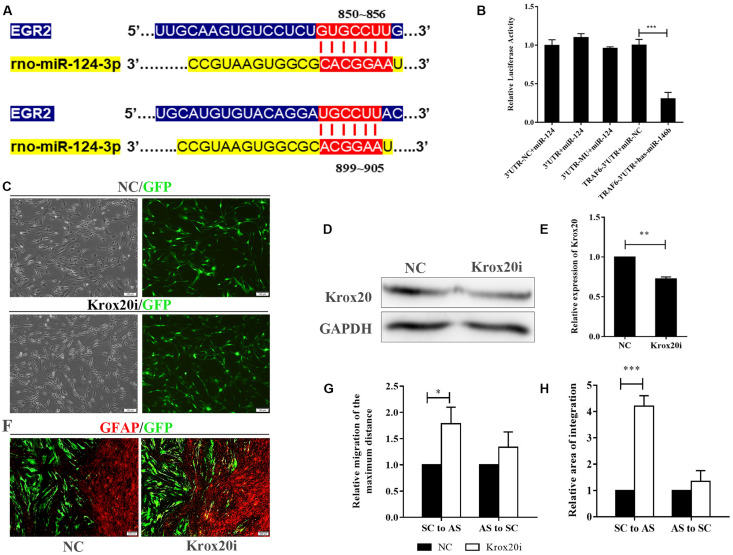
MiR-124 overexpression enhanced SC-AS integration through indirect regulation of Krox20. **(A)** Bioinformatic websites predicted that *Krox20* (*Egr2*) and *rno-miR-124-3p* had two binding sites at 850 ∼ 856 and 899 ∼ 905. **(B)** MiR-124 did not bind to 3′UTR of *Krox20* according to the dual luciferase assay. Positive control (*TRAF6-3*′*UTR* + *has-miR-146b*) showed no problems in the detection system (*n* = 3, *p* > 0.05, ***p < 0.001). **(C)** Lentivirus of Krox20-interference vectors (Krox20i) were applied to knockdown Krox20 expression in SCs (scale bar = 100 μm). **(D,E)** The efficiency of interference was analyzed by western blotting, and the results showed that the Krox20 expression was down-regulated by Krox20i (*n* = 3, **p < 0.01). **(F)** The SC-AS integration was observed by immunofluorescence. The results showed the increased ability of SC-AS integration after Krox20 was down-regulated (scale bar = 100 μm). **(G)** The relative migration of the maximum distance of SCs into the astrocytic region was higher after Krox20 interference in SCs (*n* = 3, *p < 0.05). The migration of ASs was not affected. **(H)** The relative migration area of SCs was significantly higher after Krox20 interference in SCs (*n* = 3, ***p < 0.001). The integration ability of ASs did not significantly change.

Two binding sites (850 ∼ 856 and 899 ∼ 905) between *rno-miR-124-3p* and UTR of *Krox20* have been predicted^2^ (Figure 5A). Dual luciferase assay revealed the opposite—the relative luciferase activity of *3*′*UTR-NC* + *miR-124* did not significantly change compared with that of *3*′*UTR-miR-124* (*p* > 0.05). Compared with *TRAF6-3*′*UTR* + *miR-NC*, the relative expression of luciferase in *TRAF6-3*′*UTR* + *has-miR-146b* was significantly decreased, indicating that the transfection method was successful. These results indicate that Krox20 is not a target gene of miR-124 (Figure 5B; ^∗∗∗^*p* < 0.001). We then speculated that miR-124 might have an indirect effect on Krox20 in regulating cell integration, and this mechanism remains to be studied.

To verify that the intermingling of miR-124-SCs and ASs was regulated by Krox20, we inhibited the Krox20 expression in SCs using an RNAi lentivirus (herein, Krox20i). SCs were transfected for 48 h, and green fluorescence was detected (Figure 5C). From Figures 5D,E (^∗∗^*p* < 0.01), the protein expression of Krox20 was reduced threefold as compared with the NC (examined by western blotting). Meanwhile, the boundary assay demonstrated that integration of SC-AS was promoted when Krox20 expression in SCs was affected (Figures 5F–H; ^∗^*p* < 0.05, ^∗∗∗^*p* < 0.001). Overall, these results indicate that miR-124 overexpression in SCs enhanced SC-AS integration by indirect inhibition of Krox20 expression in SCs; this regulatory mechanism needs to be further explored.

### Exploring Potential Target Genes and Signaling Pathways Involved in the Motility and Migration of Schwann Cell After MicroRNA-124 Overexpression

**FIGURE 6:**
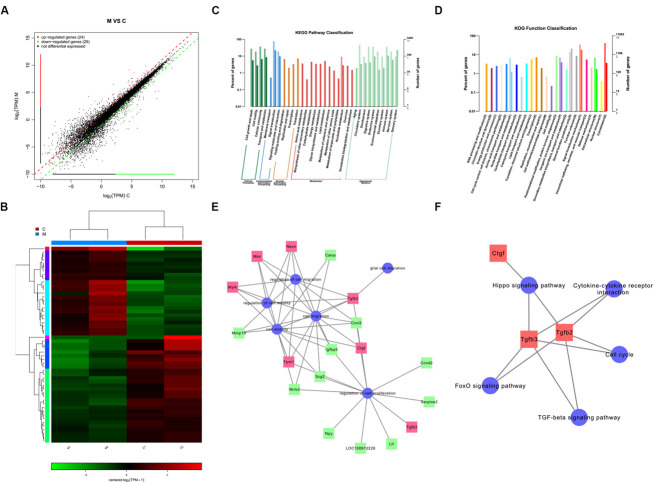
Analysis of genes and signaling pathways involved in Schwann cells (SCs) migration after overexpression of miR-124 according to RNA sequencing. **(A)** Scatter plot of differentially expressed genes. The horizontal and vertical axes are the two sets of sample log (TPM) values; each point in the figure represents a gene. Red, green, and black indicate the up-regulated, down-regulated, and non-differentiated genes, respectively. **(B)** Heatmap of differentially expressed genes. Red indicates higher gene expression level in the sample, whereas green indicates the opposite. The results showed the expression level and patterns of miR-124-SCs (M) significantly changed compared with the control group (C). **(C)** KEGG pathway classification. **(D)** KOG function classification. **(E)** GO map for genes enriched in cell motility and migration, as well as the regulation of these two processes and of cell proliferation and glial cell migration. **(F)** Pathway map for the potential molecular mechanisms and signaling pathways.

We identified a total of 24 up-regulated and 29 down-regulated genes (Figure 6A). The expression of DEGs were presented as volcano plots, and the significant differences between M and C were observed (Figure 6B). KEGG pathway (Figure 6C) and KOG function classifications (Figure 6D) were studied, and gene enrichment analysis was performed. We obtained 7 up-regulated DEG genes (Figure 6E) mainly related to cell motility according to KEGG pathway and KOG function classifications, including nexilin (*Nexn*) (Li et al., 2018), moesin (*Msn*) (Vitorino et al., 2015), myosin light chain kinase (*Mylk*) (Xia et al., 2019), connective tissue growth factor (*CTGF*) (Han et al., 2016), transforming growth factor-beta 2 (*TGF-*β*2*) (Kim et al., 2016), *transforming growth factor-beta 3* (*TGF-*β*3*) (Ju et al., 2018), and tropomyosin 1 (*Tpm1*) (Jiang et al., 2017). We found that 11 genes were down-regulated (Figure 6E), including matrix metallopeptidase 10 (*Mmp10*), calcitonin-related polypeptide alpha (*Calca*), Neurotrophic receptor tyrosine kinase (*Ntrk2*), secretogranin 2 (*Scg2*), c-x-c motif chemokine ligand-2 (*Cxcl2*), neuropeptide Y (*Npy*), insulin-like growth factor binding protein 5 (*Igfbp5*), cyclin D2 (*Ccnd2*), leukemia inhibitory factor (*Lif*), serpin family E member 2 (*Serpine 2*), and pro-neuropeptide Y-like (*LOC100912228*). The potential pathways and related mechanisms from the pathway maps included Hippo signaling pathway (Ma et al., 2017), cytokine-cytokine receptor interaction (Liu et al., 2017), FoxO signaling pathway (Hu et al., 2017), TGF-beta signaling pathway (Larco et al., 2018), and cell cycle (Bendris et al., 2015) (Figure 6F).

### Inhibition of Astrogliosis Through Negative Regulation of GFAP and p-STAT3 by MicroRNA-124 Overexpression in Schwann Cells

**FIGURE 7:**
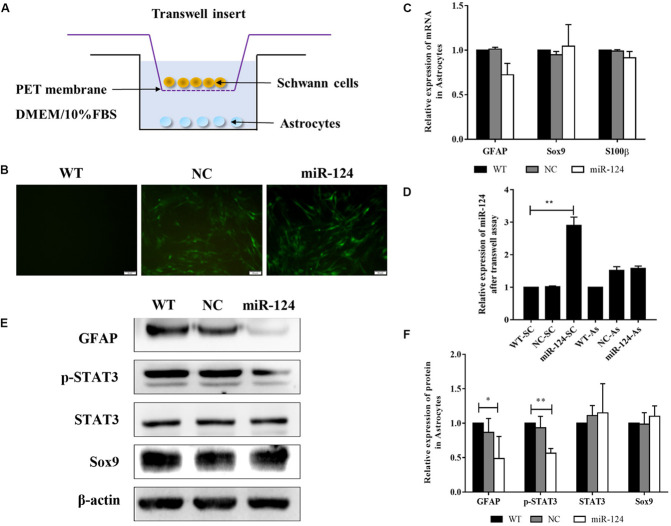
Schwann cells (SCs) inhibited astrogliosis by miR-124-mediated negative regulation of GFAP and p-STAT3. **(A)** Stereoscopic culture pattern of SCs transplanted into astrocytic environment. **(B)** SCs overexpressing miR-124 and cultured in the upper inserts emitted spontaneous green fluorescence for 2 weeks (scale bar = 100 μm). **(C)** Gene expressions of *GFAP*, *Sox9*, and *S100*β were detected by qRT-PCR in ASs; the results showed no significant change (*n* = 3, *p* > 0.05). **(D)** The relative expression of miR-124 in SCs and ASs was detected by qRT-PCR in the Transwell system. The results indicated that miR-124 expression increased in SCs but was not significantly changed in ASs (*n* = 3, **p < 0.01). **(E,F)** The protein expressions of GFAP and p-STAT3 were significantly down-regulated in ASs in the Transwell system, whereas that of Sox9 did not change (*n* = 3, *p < 0.05, **p < 0.01).

To identify the indirect effects of miR-124-SCs on ASs, a 3D-model simulating a transplantation microenvironment was established with a Transwell assay, as shown in Figure 7A. After 7 days of culture, GFP fluorescence was still steadily observed in SCs, indicative of transfected SCs (Figure 7B). The RNA expressions of *GFAP*, *Sox9*, and *S100*β in ASs did not significantly change according to qRT-PCR (Figure 7C; *p* > 0.05). JAK/STAT3 activation is critical for reactive astrogliosis and glial scar formation (Reichenbach et al., 2019). Our results indicated that the expression of GFAP and p-STAT3 protein significantly decreased in ASs after indirect co-culture with miR-124-SCs. Therefore, SCs could inhibit glial scar formation by paracrine or other mechanisms after miR-124 overexpression (Figures 7E,F; ^∗^*p* < 0.05, ^∗∗^*p* < 0.01).

To explore whether miR-124 affected the translation of GFAP and p-STAT3 in ASs under a paracrine mechanism, miR-124 expressions in SCs and ASs were analyzed in the Transwell system by qRT-PCR. Its expression in SCs was significantly higher in the experimental group, compared to those in NC and WT. Meanwhile, that in ASs was not affected (Figure 7D; ^∗∗^*p* < 0.01). These results suggested that the inhibition of GFAP and p-STAT3 expression in ASs by miR-124-SCs was not caused by the paracrine transfer of miR-124 to ASs via SC, and its mechanism needs further investigation.

## Discussion

Although its effectiveness and safety have improved, many challenges regarding SC transplantation remain. For instance, most SCs surrounded by reactive ASs remain in the lesion core with minimal capacity to migrate to the surrounding tissue following injection into the damaged spinal cord. ASs respond to CNS disorders through a process called reactive astrogliosis, which further contributes to glial scar formation. Inhibitors are produced by the glial scar, and more extracellular matrix is secreted by reactive ASs after contact with SCs, thus excluding SCs from the CNS, which limits the function of SCs in neural regeneration and repair. Therefore, we aimed to modify the bioactive characteristics and phenotype of SCs to improve their migration and integration with ASs, thus allowing SCs survival and facilitating neural tissue repair in the host spine. By inhibiting the formation of glial scar or the generation of A1-neurotoxic ASs, a permissive niche for spine regeneration could be created (Lukovic et al., 2015; Liddelow et al., 2017).

Here, the results indicated that the expression of miR-124 in SCs after transfection was increased and sustained without affecting cell proliferation. The expression of GFAP was significantly down-regulated at the gene and protein levels. GFAP is expressed by immature SCs and mature, non-myelinating SCs (Mirsky et al., 2008), and the overexpression of miR-124 probably changed SCs into mature phenotype. The expression of Sox10 was also down-regulated at the mRNA level without significant down-regulation of protein expression, presumably due to the high expression of Sox10 at the transcriptional level for a short time without affecting protein translation. Sox10 and Krox20 are closely related to the development of SCs and can synergistically regulate their myelination. These results indicated that the myelination process might be influenced by the up-regulation of miR-124. We will perform experiments on myelination formation to validate this speculation. Interestingly, we detected that the gene expressions of *Nt-3* and *Bdnf* were significantly up-regulated, thus the expression of genes related to the growth of neurons was also increased. Accordingly, we speculate that the change in the gene expression of neurotrophic factors in miR-124-SCs might ameliorate the microenvironment of SCI, thus favoring neuron survival and axon regeneration after miR-124-SC transplantation. We also plan to further investigate these changes in neurotrophic factors through ELISA. These results showed that mature miR-124 may alter the characteristics of SCs by inhibiting the expression of multiple potential target genes and affecting the myelination and the production of neurotrophic factors of SCs.

MiRNAs can regulate SC migration and proliferation through target genes (Sohn and Park, 2018); thus, we hypothesized that miR-124 altered the motility of SCs through related genes and simultaneously affected the expression of cell surface molecules and extracellular signaling to enhance SC migration. MiRNAs have been previously shown to suppress SC proliferation and migration by targeting *Bdnf* (Yi et al., 2016). However, in the present study, the expression of *Bdnf* and *Nt-3* genes was significantly up-regulated in miR-124-SCs, which might be the reason for the increased SC motor ability. Furthermore, nutrient factors might regulate cell surface molecules and bind cell-specific receptors (Yamauchi et al., 2004), thus modulating the movement of SCs and ASs via miR-124 signaling network. We further noted that the expressions of EphA4 and N-Cadherin proteins in miR-124-SCs did not significantly change, whereas that of Krox20 was clearly down-regulated, which is in agreement with a recent study showing that the invasion and migration of ASs and oligodendrocytes into the PNS region occurred after Krox20 inactivation (Coulpier et al., 2010). In the present study, we validated that interference with Krox20 expression in SCs significantly enhanced the SC-AS integration. Therefore, the down-regulation of Krox20 was induced by miR-124-enhanced SC migration. In other words, miR-124 enhanced the movement of both SCs and ASs through more complex, unelucidated mechanisms.

To further explore potential target genes and signaling pathways involved in SC motility and migration after miR-124 overexpression, we analyzed the differentially expressed genes and signaling pathways using RNA-seq technology and several databases. According to the heat map and scatter plot analyses of DEGs, 24 genes were up-regulated, and 29 were down-regulated in miR-124-SCs, of which 7 up-regulated and 11 down-regulated DEGs were related to promote migration and motility of cells. Among these, we focused on Sox10 as the leading cause of the alteration of SC traits after miR-124 overexpression. Sox10 has been reported to be expressed throughout the development of SCs as it is essential for their specification and identification. *Sox10* deletion affected the integrity of the CNS/PNS boundary function, and the expression of *Sox10* was under the control of *Krox20* regulatory elements (Frob et al., 2012). In our experiments, the transcription of Sox10 was down-regulated in SCs after miR-124 overexpression, whereas its expression Krox20 remained remarkably inhibited, which promoted the SC-AS integration. Furthermore, RNA-seq technology showed that the expression of *TGF-*β (*TGF-*β*2* and *TGF-*β*3*) was up-regulated in the miR-124-SCs. Neural crest stem cells can generate mesenchymal progenitors by the negative regulation of *Sox10* via TGF-β signaling pathway (John et al., 2011). Thus, we speculated that the enhancement of SC-AS integration might be negatively regulated by the Krox20/Sox10 signaling through TGF-β pathway after miR-124 overexpression in SCs.

Furthermore, we hypothesized that miR-124 overexpression in SCs indirectly restrained the glial properties and activation of ASs. Our results showed that the protein expression levels of GFAP and p-STAT3 significantly decreased in ASs after their co-culture with miR-124-SCs in the Transwell system. GFAP is the marker of reactive ASs, and thus becomes intensely positive after damage. The active proliferation of reactive ASs depended on the activation of the STAT3 signaling pathway (Zhou et al., 2017). Our results illustrated that miR-124-SCs could inhibit the protein expressions of GFAP and p-STAT3 in ASs, suggesting that miR-124-SCs may repress AS reactivity and the formation of glial scar. Because we used a Transwell culture system, in which the two type of cells did not interact directly, we conclude that miR-124-SCs indirectly influenced the characteristics of ASs by secreting biological factors including growth factors and cytokines. As mentioned above, after miR-124 overexpression in SCs, the mRNA expression levels of *Nt-3* and *Bdnf* were up-regulated, indicating that, at the least, miR-124-SCs might increase the secretion of these neurotrophic factors and influence the phenotypes of ASs after their co-culture with miR-124-SCs.

In summary, miR-124 overexpression in SCs affected SCs characteristics and promoted its integration with ASs through the inhibition of Krox20 expression via indirect regulation of miR-124. The reactive activity of ASs was restrained by miR-124-SCs, which may reduce glial scar formation. To further elucidate the integration of transplant miR-124-SC graft with the host ASs, we plan to transplant SC graft into rat spinal cord after SCI surgery in the future. Thus, our novel findings expanded the therapeutic potential of SC transplantation following SCI.

## Data Availability Statement

The sequencing data for this study can be found in NCBI (https://www.ncbi.nlm.nih.gov/Traces/study1/?acc=SRP250133 +&go=go).

## Ethics Statement

The animal study was reviewed and approved by the Ethics Committee of Jilin University.

## Author Contributions

YH and GC designed the research. ZL, YH, and GC wrote the manuscript. ZL, YY, JK, YZ, JX, KX, and KH performed the experiments and analyzed the data. ZL, YY, JK, YH, and GC reviewed and edited the manuscript. All authors have read and approved the final manuscript.

## Conflict of Interest

The authors declare that the research was conducted in the absence of any commercial or financial relationships that could be construed as a potential conflict of interest.

## Footnotes

1 www.targetscan.org

2 www.targetscan.org

## Abbreviations

AS: astrocyte
AsM: astrocyte culturing medium
BDNF: brain-derived neurotrophic factor
CNS: central nervous system
CNTF: ciliary neurotrophic factor
DMEM: Dulbecco’s modified eagle medium
EGR2 (also known as Krox20): early growth response 2
FBS: fetal bovine serum
GFAP: glial fibrillary acidic protein
GFP: green fluorescent protein
miR-124: microRNA-124
miR-124-SC: Schwann cell overexpressing miR-124
miRNA: microRNA
MOI: multiplicity of infection
NC: negative control
NGF: nerve growth factor
Nt-3: neurotrophin 3
PBS: phosphate buffer saline
PCR: polymerase chain reaction
PFA: paraformaldehyde
PNS: peripheral nervous system
p-STAT 3: phosphorylated-signal transducer and activator of transcription 3
RT: reverse transcription
SC: Schwann cell
SCI: spinal cord injury
ScM: Schwann cell culturing medium
Sox10: SRY-Box transcription factor 10
Sox9: SRY-Box transcription factor 9
STAT3: signal transducer and activator of transcription 3
TGF- β: transforming growth factor- β
WT: wild type.

## References

[B1] Afshari F. T. Fawcett J. W. (2012). Astrocyte-schwann-cell coculture systems. *Methods Mol. Biol.* 814 381–391. 10.1007/978-1-61779-452-0_25 22144320

[B2] Afshari F. T. Kwok J. C. Fawcett J. W. (2010). Astrocyte-produced ephrins inhibit schwann cell migration via VAV2 signaling. *J. Neurosci.* 30 4246–4255. 10.1523/JNEUROSCI.3351-09.2010 20335460 PMC6634495

[B3] Afshari F. T. Kwok J. C. Fawcett J. W. (2011). Analysis of schwann-astrocyte interactions using in vitro assays. *J. Vis. Exp.* 47:2214. 10.3791/2214 21304451 PMC3182651

[B4] Ambasudhan R. Talantova M. Coleman R. Yuan X. Zhu S. Lipton S. A. (2011). Direct reprogramming of adult human fibroblasts to functional neurons under defined conditions. *Cell Stem Cell* 9 113–118. 10.1016/j.stem.2011.07.002 21802386 PMC4567246

[B5] Andersen N. D. Srinivas S. Pinero G. Monje P. V. (2016). A rapid and versatile method for the isolation, purification and cryogenic storage of schwann cells from adult rodent nerves. *Sci. Rep.* 6:31781. 10.1038/srep31781 27549422 PMC4994039

[B6] Anderson K. D. Guest J. D. Dietrich W. D. Bartlett Bunge M. Curiel R. Dididze M. (2017). Safety of autologous human schwann cell transplantation in subacute thoracic spinal cord injury. *J. Neurotraum.* 34 2950–2963. 10.1089/neu.2016.4895 28225648

[B7] Andrews M. R. Stelzner D. J. (2007). Evaluation of olfactory ensheathing and schwann cells after implantation into a dorsal injury of adult rat spinal cord. *J. Neurotraum.* 24 1773–1792. 10.1089/neu.2007.0353 18001205

[B8] Assinck P. Duncan G. J. Hilton B. J. Plemel J. R. Tetzlaff W. (2017). Cell transplantation therapy for spinal cord injury. *Nat. Neurosci.* 20 637–647. 10.1038/nn.4541 28440805

[B9] Bendris N. Lemmers B. Blanchard J. M. (2015). Cell cycle, cytoskeleton dynamics and beyond: the many functions of cyclins and CDK inhibitors. *Cell Cycle* 14 1786–1798. 10.1080/15384101.2014.998085 25789852 PMC4614797

[B10] Blakemore W. F. Crang A. J. Curtis R. (1986). The interaction of schwann cells with CNS axons in regions containing normal astrocytes. *Acta Neuropathol.* 71 295–300. 10.1007/bf00688052 3799142

[B11] Coulpier F. Decker L. Funalot B. Vallat J. M. Garcia-Bragado F. Charnay P. (2010). CNS/PNS boundary transgression by central glia in the absence of schwann cells or Krox20/Egr2 function. *J. Neurosci.* 30 5958–5967. 10.1523/JNEUROSCI.0017-10.2010 20427655 PMC6632613

[B12] Fairless R. Frame M. C. Barnett S. C. (2005). N-cadherin differentially determines schwann cell and olfactory ensheathing cell adhesion and migration responses upon contact with astrocytes. *Mol. Cell Neurosci.* 28 253–263. 10.1016/j.mcn.2004.09.009 15691707

[B13] Fan B. Wei Z. Yao X. Shi G. Cheng X. Zhou X. (2018). Microenvironment imbalance of spinal cord injury. *Cell Transplant* 27 853–866. 10.1177/0963689718755778 29871522 PMC6050904

[B14] Faul F. Erdfelder E. Lang A. G. Buchner A. (2007). G^∗^Power 3: a flexible statistical power analysis program for the social, behavioral, and biomedical sciences. *Behav. Res. Methods* 39 175–191. 10.3758/bf03193146 17695343

[B15] Faulkner J. R. Herrmann J. E. Woo M. J. Tansey K. E. Doan N. B. Sofroniew M. V. (2004). Reactive astrocytes protect tissue and preserve function after spinal cord injury. *J. Neurosci.* 24 2143–2155. 10.1523/JNEUROSCI.3547-03.2004 14999065 PMC6730429

[B16] Frob F. Bremer M. Finzsch M. Kichko T. Reeh P. Tamm E. R. (2012). Establishment of myelinating schwann cells and barrier integrity between central and peripheral nervous systems depend on Sox10. *Glia* 60 806–819. 10.1002/glia.22310 22337526

[B17] Gu Y. Chen C. Yi S. Wang S. Gong L. Liu J. (2015). miR-sc8 inhibits schwann cell proliferation and migration by targeting egfr. *PLoS One* 10:e0145185. 10.1371/journal.pone.0145185 26683191 PMC4686161

[B18] Han Q. Zhang H. Y. Zhong B. L. Wang X. J. Zhang B. Chen H. (2016). MicroRNA-145 inhibits cell migration and invasion and regulates epithelial-mesenchymal transition (EMT) by targeting connective tissue growth factor (CTGF) in esophageal squamous cell carcinoma. *Med. Sci. Monit.* 22 3925–3934. 10.12659/msm.897663 27771733 PMC5081241

[B19] Hu Q. Wang G. Peng J. Qian G. Jiang W. Xie C. (2017). Knockdown of SIRT1 suppresses bladder cancer cell proliferation and migration and induces cell cycle arrest and antioxidant response through FOXO3a-mediated pathways. *Biomed. Res. Int.* 2017:3781904. 10.1155/2017/3781904 29147649 PMC5632854

[B20] Jessen K. R. Mirsky R. Lloyd A. C. (2015). Schwann cells: development and role in nerve repair. *Cold Spring Harb. Perspect. Biol.* 7:a020487. 10.1101/cshperspect.a020487 25957303 PMC4484967

[B21] Jiang R. Zhang C. Liu G. Gu R. Wu H. (2017). MicroRNA-107 promotes proliferation, migration, and invasion of osteosarcoma cells by targeting tropomyosin 1. *Oncol. Res.* 25 1409–1419. 10.3727/096504017X14882829077237 28276320 PMC7841194

[B22] John N. Cinelli P. Wegner M. Sommer L. (2011). Transforming growth factor beta-mediated Sox10 suppression controls mesenchymal progenitor generation in neural crest stem cells. *Stem Cells* 29 689–699. 10.1002/stem.607 21308864

[B23] Ju Q. Li M. X. Chen G. Wang H. X. Shi Q. M. Ge X. (2018). Overexpression of YOD1 promotes the migration of human oral keratinocytes by enhancing TGF-beta3 signaling. *Biomed. Environ. Sci.* 31 499–506. 10.3967/bes2018.067 30145984

[B24] Kim S. Han J. Jeon M. You D. Lee J. Kim H. J. (2016). Silibinin inhibits triple negative breast cancer cell motility by suppressing TGF-beta2 expression. *Tumour. Biol.* 37 11397–11407. 10.1007/s13277-016-5000-5007 26984157

[B25] Larco D. O. Bauman B. M. Cho-Clark M. Mani S. K. Wu T. J. (2018). GnRH-(1-5) inhibits TGF-beta signaling to regulate the migration of immortalized gonadotropin-releasing hormone neurons. *Front. Endocrinol.* 9:45. 10.3389/fendo.2018.00045 29515521 PMC5826220

[B26] Li Q. Zhao H. Pan P. Ru X. Zuo S. Qu J. (2018). Nexilin regulates oligodendrocyte progenitor cell migration and remyelination and is negatively regulated by protease-activated receptor 1/Ras-proximate-1 signaling following subarachnoid hemorrhage. *Front. Neurol.* 9:282. 10.3389/fneur.2018.00282 29922213 PMC5996890

[B27] Li S. Yue Y. Xu W. Xiong S. (2013). MicroRNA-146a represses mycobacteria-induced inflammatory response and facilitates bacterial replication via targeting IRAK-1 and TRAF-6. *PLoS One* 8:e81438. 10.1371/journal.pone.0081438 24358114 PMC3864784

[B28] Liddelow S. A. Guttenplan K. A. Clarke L. E. Bennett F. C. Bohlen C. J. Schirmer L. (2017). Neurotoxic reactive astrocytes are induced by activated microglia. *Nature* 541 481–487. 10.1038/nature21029 28099414 PMC5404890

[B29] Lim L. P. Garrett-Engele P. Grimson A. Schelter J. M. Castle J. Bartel D. P. (2005). Microarray analysis shows that some microRNAs downregulate large numbers of target mRNAs. *Nature* 433 769–773. 15685193 10.1038/nature03315

[B30] Lin R. Z. Moreno-Luna R. Zhou B. Pu W. T. Melero-Martin J. M. (2012). Equal modulation of endothelial cell function by four distinct tissue-specific mesenchymal stem cells. *Angiogenesis* 15 443–455. 10.1007/s10456-012-9272-9272 22527199 PMC3409933

[B31] Liu W. Liu D. Zheng J. Shi P. Chou P. H. Oh C. (2017). Annulus fibrosus cells express and utilize C-C chemokine receptor 5 (CCR5) for migration. *Spine J.* 17 720–726. 10.1016/j.spinee.2017.01.010 28108404 PMC5673099

[B32] Lukovic D. Stojkovic M. Moreno-Manzano V. Jendelova P. Sykova E. Bhattacharya S. S. (2015). Concise review: reactive astrocytes and stem cells in spinal cord injury: good guys or bad guys? *Stem Cells* 33 1036–1041. 10.1002/stem.1959 25728093

[B33] Ma X. Wang H. Ji J. Xu W. Sun Y. Li W. (2017). Hippo signaling promotes JNK-dependent cell migration. *Proc. Natl. Acad. Sci. U.S.A.* 114 1934–1939. 10.1073/pnas.1621359114 28174264 PMC5338425

[B34] Mirsky R. Woodhoo A. Parkinson D. B. Arthur-Farraj P. Bhaskaran A. Jessen K. R. (2008). Novel signals controlling embryonic Schwann cell development, myelination and dedifferentiation. *J. Peripher. Nerv. Syst.* 13 122–135. 10.1111/j.1529-8027.2008.00168.x 18601657

[B35] Mondanizadeh M. Arefian E. Mosayebi G. Saidijam M. Khansarinejad B. Hashemi S. M. (2015). MicroRNA-124 regulates neuronal differentiation of mesenchymal stem cells by targeting Sp1 mRNA. *J. Cell Biochem.* 116 943–953. 10.1002/jcb.25045 25559917

[B36] O’Shea T. M. Burda J. E. Sofroniew M. V. (2017). Cell biology of spinal cord injury and repair. *J. Clin. Invest.* 127 3259–3270. 10.1172/JCI90608 28737515 PMC5669582

[B37] Park H. Huang X. Lu C. Cairo M. S. Zhou X. (2015). MicroRNA-146a and microRNA-146b regulate human dendritic cell apoptosis and cytokine production by targeting TRAF6 and IRAK1 proteins. *J. Biol. Chem.* 290 2831–2841. 10.1074/jbc.M114.591420 25505246 PMC4317016

[B38] Pearse D. D. Sanchez A. R. Pereira F. C. Andrade C. M. Puzis R. Pressman Y. (2007). Transplantation of schwann cells and/or olfactory ensheathing glia into the contused spinal cord: survival, migration, axon association, and functional recovery. *Glia* 55 976–1000. 10.1002/glia.20490 17526000

[B39] Reichenbach N. Delekate A. Plescher M. Schmitt F. Krauss S. Blank N. (2019). Inhibition of Stat3-mediated astrogliosis ameliorates pathology in an Alzheimer’s disease model. *EMBO Mol. Med.* 11:1809665. 10.15252/emmm.201809665 30617153 PMC6365929

[B40] Reiprich S. Kriesch J. Schreiner S. Wegner M. (2010). Activation of Krox20 gene expression by Sox10 in myelinating schwann cells. *J. Neurochem.* 112 744–754. 10.1111/j.1471-4159.2009.06498.x 19922439

[B41] Saberi H. Firouzi M. Habibi Z. Moshayedi P. Aghayan H. R. Arjmand B. (2011). Safety of intramedullary Schwann cell transplantation for postrehabilitation spinal cord injuries: 2-year follow-up of 33 cases. *J. Neurosurg. Spine.* 15 515–525. 10.3171/2011.6.SPINE10917 21800956

[B42] Shields S. A. Blakemore W. Franklin R. J. (2000). Schwann cell remyelination is restricted to astrocyte-deficient areas after transplantation into demyelinated adult rat brain. *J. Neurosci.* 60 571–578. 10.1002/(sici)1097-4547(20000601)60:5<571::aid-jnr1>3.0.co;2-q 10820427

[B43] Silver J. (2016). The glial scar is more than just astrocytes. *Exp. Neurol.* 286 147–149. 10.1016/j.expneurol.2016.06.018 27328838

[B44] Sofroniew M. V. Vinters H. V. (2010). Astrocytes: biology and pathology. *Acta Neuropathol.* 119 7–35. 10.1007/s00401-009-0619-618 20012068 PMC2799634

[B45] Sohn E. J. Park H. T. (2018). MicroRNA mediated regulation of schwann cell migration and proliferation in peripheral nerve injury. *Biomed. Res. Int.* 2018:8198365. 10.1155/2018/8198365 29854793 PMC5952561

[B46] Sun Y. Luo Z. M. Guo X. M. Su D. F. Liu X. (2015). An updated role of microRNA-124 in central nervous system disorders: a review. *Front. Cell Neurosci.* 9:193. 10.3389/fncel.2015.00193 26041995 PMC4438253

[B47] Vitorino P. Yeung S. Crow A. Bakke J. Smyczek T. West K. (2015). MAP4K4 regulates integrin-FERM binding to control endothelial cell motility. *Nature* 519 425–430. 10.1038/nature14323 25799996

[B48] Xia B. Huang L. Zhu L. Liu Z. Ma T. Zhu S. (2016). Manipulation of Schwann cell migration across the astrocyte boundary by polysialyltransferase-loaded superparamagnetic nanoparticles under magnetic field. *Int. J. Nanomed.* 11 6727–6741. 10.2147/IJN.S122358 28003748 PMC5161335

[B49] Xia N. Cui J. Zhu M. Xing R. Lu Y. (2019). Androgen receptor variant 12 promotes migration and invasion by regulating MYLK in gastric cancer. *J. Pathol.* 248 304–315. 10.1002/path.5257 30737779

[B50] Yamauchi J. Chan J. Shooter E. M. (2004). Neurotrophins regulate schwann cell migration by activating divergent signaling pathways dependent on Rho GTPases. *Proc. Natl. Acad. Sci. U.S.A.* 101 8774–8779. 10.1073/pnas.0402795101 15161978 PMC423271

[B51] Yi S. Yuan Y. Chen Q. Wang X. Gong L. Liu J. (2016). Regulation of Schwann cell proliferation and migration by miR-1 targeting brain-derived neurotrophic factor after peripheral nerve injury. *Sci. Rep.* 6:29121. 10.1038/srep29121 27381812 PMC4933896

[B52] Yu B. Zhou S. Wang Y. Qian T. Ding G. Ding F. (2012). miR-221 and miR-222 promote schwann cell proliferation and migration by targeting LASS2 after sciatic nerve injury. *J. Cell Sci.* 125 (Pt 11), 2675–2683. 10.1242/jcs.098996 22393241

[B53] Zhou H. J. Yang X. Cui H. J. Tang T. Zhong J. H. Luo J. K. (2017). Leukemia inhibitory factor contributes to reactive astrogliosis via activation of signal transducer and activator of transcription 3 signaling after intracerebral hemorrhage in rats. *J. Neurotraum.* 34 1658–1665. 10.1089/neu.2016.4711 27825285

